# Interleukin-27 and Autoimmune Disorders: A Compressive Review of Immunological Functions

**DOI:** 10.3390/biom14121489

**Published:** 2024-11-22

**Authors:** Esmaeil Yazdanpanah, Alireza Pazoki, Sepehr Dadfar, Mohammad Hosein Nemati, Seyed Mohammad Sajad Siadati, Mahdieh Tarahomi, Niloufar Orooji, Dariush Haghmorad, Valentyn Oksenych

**Affiliations:** 1 Student Research Committee, Semnan University of Medical Sciences, Semnan 35147-99442, Iran; 2 Department of Immunology, School of Medicine, Semnan University of Medical Sciences, Semnan 35147-99442, Iran; 3 Department of Clinical and Molecular Medicine, Norwegian University of Science and Technology (NTNU), 7028 Trondheim, Norway; 4 Department of Biosciences and Nutrition, Karolinska Institutet, 14183 Huddinge, Sweden

**Keywords:** autoimmune disorders, interleukin-27, multiple sclerosis, rheumatoid arthritis, type 1 diabetes, inflammatory bowel disease

## Abstract

Autoimmune disorders (ADs) pose significant health and economic burdens globally, characterized by the body’s immune system mistakenly attacking its own tissues. While the precise mechanisms driving their development remain elusive, a combination of genetic predisposition(s) and environmental triggers is implicated. Interleukin-27 (IL-27), among numerous cytokines involved, has emerged as a key regulator, exhibiting dual roles in immune modulation. This review delves into the molecular structure and signaling mechanisms of IL-27, highlighting its diverse effects on various immune cells. Additionally, it explores the involvement of IL-27 in autoimmune diseases, such as multiple sclerosis (MS) and rheumatoid arthritis (RA), offering insights into its potential therapeutic implications. Moreover, its involvement in autoimmune diseases like type 1 diabetes (T1D), inflammatory bowel disease (IBD), myasthenia gravis (MG), Sjögren’s syndrome (SS), and Guillain-Barré syndrome (GBS) is multifaceted, with potential diagnostic and therapeutic implications across these conditions. Further research is essential to fully understand IL-27’s mechanisms of action and therapeutic potential in autoimmune diseases.

## 1. Introduction

Autoimmune disorders (ADs) are characterized as a condition in which the body’s immune system attacks self-antigens as a result of a failure or breakdown of immunological tolerance [1]. The underlying nature of the development of autoimmunity and ADs remains largely unknown, but ADs, like many other complex disorders, are believed to arise from a combination of genetic predisposition(s) and environmental triggers [2]. ADs have dramatically increased over recent decades, from an estimated prevalence of 4.5%, with 2.7% for males and 6.4% for females [3]. It is also important to note that ADs are associated with major societal and economic consequences due to a lack of effective treatments [4]. There are more than 80 types of autoimmune disorders, which are generally divided into tissue-specific, which affect particular targets in the body (autoimmune thyroid disease, type 1 diabetes, psoriasis, multiple sclerosis, and Guillain–Barré syndrome), or systemic, which engage multiple organs (systemic lupus erythematosus, rheumatoid arthritis, chronic inflammatory bowel disease, Sjögren’s syndrome, and antiphospholipid syndrome [5].

Immunologically, autoimmune diseases appear to be driven by dysregulation of the effector and regulatory immune responses, leading to a shift in the immune system towards a pro-inflammatory state, the production of autoantibodies, and tissue destruction [6]. The growing progress in cellular and molecular biology has revealed that helper T cells play various essential roles in the development and maintenance of various autoimmune diseases [7]. Although Th1 and Th17 play a deleterious role in the initiation and development of ADs, Th2 and Treg cells limit pathogenic autoimmune responses [7].

Mounting evidence has convincingly demonstrated that cytokines, as mediators of cellular and humoral immune responses, play a pivotal role in the pathogenesis of various autoimmune and other immune-mediated diseases [8]. IL-27, a heterodimeric immunological factor of the IL-12/IL-23 cytokine family, is mainly produced by activated antigen-presenting cells (APCs) [9]. Dysregulation of IL-27 concentrations and their correlation with autoimmune parameters have also been shown to relate to the immunopathogenesis of Th1/Th17-mediated inflammatory diseases, such as rheumatoid arthritis, multiple sclerosis, inflammatory bowel disease, and Behçet’s disease (BD) [10,11,12,13,14].

Initially, IL-27 was predicted to be an immune-enhancing and pro-inflammatory cytokine due to its ability to promote Th1 differentiation and increased IFN-γ production [9,15]. However, subsequent studies have shown the diverse functions of IL-27 in various immune responses. It also functions as an essential regulator of immune response and inflammation through the inhibition of Th2 and Th17 cell responses, the inhibition of the production of pro-inflammatory cytokines, and by inducing regulatory T cells [16,17,18,19]. Therefore, IL-27 displays dual-functioning immune regulatory effects, which may result in both immunostimulatory as well as immunosuppressive capabilities on various immune cells, depending on cell context, immune cells, and pathogen stimulus [16,17]. Together, these properties make IL-27 a potential therapeutic target in the treatment of autoimmune and inflammatory diseases. The purpose of this review is to briefly discuss the structure and function of IL-27 and highlight recent advances focusing on the roles of IL-27 in the pathogenesis of autoimmune diseases.

## 2. The Molecular Structure, Cellular Origins, and Receptor Signaling Mechanisms of IL-27

IL-27 was discovered by Pflanz and colleagues more than two decades ago as a novel cytokine, structurally and architecturally related to IL-12 and IL-23 [20]. IL-27 is a Type-I-cytokine belonging to the IL-12 cytokine family, which includes IL-12, IL-23, IL-27, IL-35, and IL-39 [21]. The heterodimeric, glycosylated protein IL-27 is composed of two subunits: a soluble receptor, Epstein–Barr virus-induced gene 3 (EBI3), and p28 (also known as IL-27 p28, IL-27A, IL-30) [9]. Increasing evidence shows an unstable linkage between p28 and EBI3 and these subunits can be released independently [9]

The IL-27 p28 subunit exhibits structural and sequence homology to IL-23 p19, IL-12 p35 and IL-6 [16,22]. EBI3, another subunit of IL-27, was first identified as being expressed in B lymphocytes infected by Epstein–Barr virus, which share sequence homology with IL-12 p40 [22]. While EBI3 is structurally similar to soluble members of the class I cytokine receptor, p28 exhibits a long-chain four-α helix bundle protein [23]. Human p28 and Ebi3 are encoded in separate genomic loci on chromosomes 16p11.2 and 19p13.3, respectively, and mouse p28 and Ebi3 on chromosomes 7qF3 and 17qD, respectively [24]. Activated APCs, such as monocytes, macrophages, and dendritic cells have been recognized as a principal cellular source of IL-27 following stimulation by microbial products or other immune stimuli, notwithstanding other cell types like endothelial cells, epithelial cells, microglia, NK cells, and plasma cells can also release lower levels of IL-27 [25,26,27]. Furthermore, it was suggested that the activation of CD40 ligation, interferon (IFN) receptors, complements toll-like receptor signaling by immune stimulators, leading to an increase in the expression of IL-27p28 and Ebi3 mRNA. This, in turn, results in an elevation in the synthesis of IL-27 by APCs [15,25,28].

IL-27 signals through a heterodimeric cell surface receptor composed of IL-27Rα (TCCR/WSX-1) and signal-transducing glycoprotein 130 (gp130) [9,27]. While gp130 is ubiquitously expressed, IL-27Rα appears to be more restricted to immune cells, such as T, B, NK, plasma, and possibly cells of the myeloid lineage [20]. Of note, the IL-27Rα expression in effector and memory T cells is high while the expression is low in naïve T cells [29].

## 3. Signaling Pathways of IL-27: From JAK/STAT Activation to Cellular Responses

Like many other cytokines, signal transduction in IL-27-mediated signaling responses has been shown to be mediated via a range of the Janus JAK/signal transducer, the activator of transcription (STAT) pathway, as well as the mitogen-activated protein kinase (MAPK) pathway, depending on the cell type [8,30]. Both IL27Rα and gp130 receptor subunits, as members of the immunoglobulin superfamily, are essential for IL-27 signal transduction and they do not signal independently [22,29]. Unlike the GP130 subunit, which is shared with IL-6 and IL-35 receptors, IL-27Rα is specific for IL-27. Therefore, the specificity of IL-27 signaling depends on IL27Rα [28,29]. The cytoplasmic region of Gp130 is related to JAK family members, JAK1, JAK2, and tyrosine kinase (TYK) 2 [27], while the cytoplasmic tail of IL-27Rα is associated with JAK1 and JAK2 and may assist in signal transduction [31]. Following receptor binding, JAK tyrosine kinases are activated, creating requisite docking sites for latent STAT monomers. Eventually, tyrosine-phosphorylated STATs dimerize, transport to the nucleus, and bind to nonameric DNA sequences known as gamma-activated sites (GAS), where they either activate or suppress gene transcription involved in multiple biological activities [28]. Therefore, when IL-27 engages its receptor, IL-27Rα, triggers the activation of the receptor-associated JAK and JAK2, which subsequently induces isoforms of STAT activation [29], which varies in a context-dependent manner, depending on cell type and activation state [32]: STAT1, STAT2, STAT3, STAT4, STAT5, JAK1, JAK2, and tyrosine kinase 2 (Tyk2) in naive CD4 T cells [33]; STAT1, STAT3, and NF-κB activation in monocytes [34]; STAT1, STAT3, STAT5, and JAK1-1 in NK cells [35,36], and STAT3 in mast cells [27].

**Figure 1:**
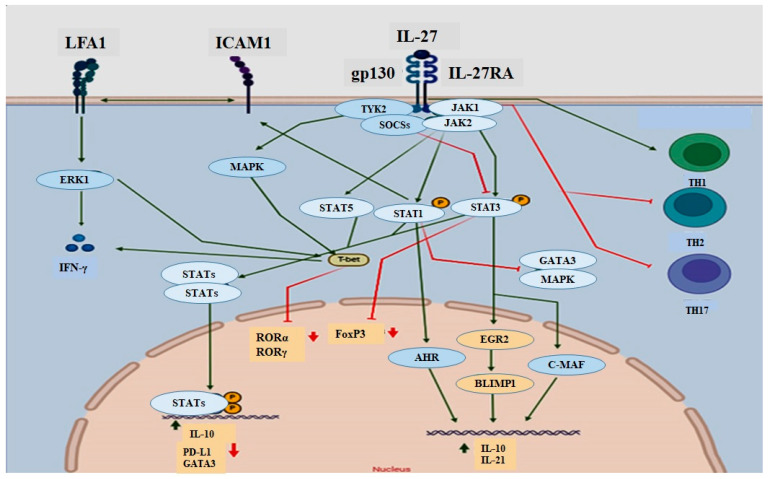
IL-27 signaling pathways. In IL-27-mediated signaling, JAK/STAT and MAP/ERK pathways play a crucial role. IL-27RA and gp130 receptor subunits are essential for IL-27 signal transduction. Upon receptor binding, JAK kinases and TYK are activated, leading to the phosphorylation of STAT proteins and the activation of the MAPK pathway. These phosphorylated STATs dimerize, translocate to the nucleus, and regulate gene transcription. IL-27-induced STAT1 and STAT3 signaling influence Th-cell differentiation as well as IL-10 and IL-21 production. Additionally, IL-27 can activate STAT2, STAT4, and STAT5 pathways. ICAM1: intercellular adhesion molecule 1; LFA1: lymphocyte function-associated antigen 1; JAK: Janus kinase; STAT: signal transducer and activator of transcription; MAPK: mitogen-activated protein kinase; ERK1: extracellular signal-regulated kinase 1; RORα: RAR-related orphan receptor alpha; RORγ: RAR-related orphan receptor gamma; PD-L1: programmed death-ligand 1; T-bet: T-box expressed in T cells; AHR: aryl hydrocarbon receptor; EGR2: early growth response protein 2; C-MAF: cellular musculoaponeurotic fibrosarcoma oncogene homolog; BLIMP1: B lymphocyte-induced maturation protein 1; TH: T helper; IFN-γ: interferon-gamma; TYK2: tyrosine kinase 2; SOCS3: suppressor of cytokine signaling 3. (Created with BioRender.com).

IL-27-induced phosphorylation of STAT1 has been shown to be involved in the inhibition of Th17 [37,38,39] and Th2 differentiation [36], whereas IL-27-induced STAT1 and STAT3 signaling have been shown to be involved in IL-10 production in T cells [40,41]. In addition, IL-27 is able to efficiently drive Th1 differentiation through two distinct pathways: ICAM-1/LFA-1 interactions in a STAT1-dependent pathway and p38 MAPK/T-bet pathways [42]. IL27 has been shown to enhance the generation of human and in mouse CD8^+^ T cells with increased granzyme B and perforin production in a STAT1-dependent [43,44]. Furthermore, the effect of IL-27 on CD8 T-cell affinity, function, expansion, and memory programming was exerted through a cascade of STAT1/3-dependent cytokines [45]. In addition to STAT1 and STAT3, IL-27 can also exert its biological properties by activating the STAT2, STAT4, and STAT5 pathways (Figure 1) [29].

## 4. The Immunomodulatory Landscape of IL-27: From Inflammation to Immune Tolerance

This IL-27 stands as a pivotal regulator within the intricate network of immune responses, exerting multifaceted effects on various immune cells. Initially identified as a product of activated antigen-presenting cells, such as macrophages and dendritic cells, IL-27 has since been recognized for its diverse roles in immune modulation, ranging from inflammation to tolerance [46].

One of the primary functions of IL-27 lies in its ability to modulate T cell responses. It exerts both pro-inflammatory and anti-inflammatory effects depending on the context of the immune microenvironment [47]. As a pro-inflammatory cytokine, IL-27 promotes the differentiation of naïve CD4^+^ T cells into pro-inflammatory Th1 cells, characterized by the production of IFN-γ and IL-2. Additionally, IL-27 inhibits the differentiation of Th17 cells, thus regulating the balance between Th1 and Th17 responses [48].

In addition to its effects on T cells, IL-27 also influences B cell function. It enhances the proliferation and differentiation of B cells, promoting the production of antibodies, particularly the IgG subclass [49]. Furthermore, IL-27 regulates the class switching of B cells, favoring the production of IgG2a and IgG2b antibodies. These antibody subclasses are crucial for effective immune responses against intracellular pathogens [50].

IL-27 exerts direct effects on innate immune cells, including NK cells and macrophages. It enhances the cytotoxic activity of NK cells, facilitating the elimination of infected or malignant cells [51]. Moreover, IL-27 promotes the polarization of macrophages towards an M1-like phenotype, characterized by increased production of pro-inflammatory cytokines and enhanced microbicidal activity. These effects contribute to the host defense against microbial pathogens and tumor cells [52].

Beyond its pro-inflammatory functions, which were mentioned above, IL-27 also plays a crucial role in immune regulation and homeostasis. It suppresses excessive inflammation by inhibiting the production of pro-inflammatory cytokines, such as IL-17 and IL-23 [53]. Additionally, IL-27 restrains the expansion and activation of effector T cells, thereby preventing immune-mediated tissue damage and autoimmunity [54]. Furthermore, IL-27 has been implicated in the induction of immune tolerance, promoting the development of regulatory T cells and dampening excessive immune responses [55].

In summary, IL-27 exerts diverse effects on immune cells, orchestrating the balance between inflammation and tolerance. Its pleiotropic functions make IL-27 an intriguing target for therapeutic intervention in various immune-mediated diseases, ranging from autoimmune disorders to cancer.

## 5. IL-27 in Multiple Sclerosis: From Pathogenesis to Therapeutic Potential

Multiple sclerosis (MS) is a chronic inflammatory disease of the central nervous system (CNS), mediated by both imbalanced T helper (Th) subsets and the disturbed cytokine profile, resulting in demyelination, axonal damage, and subsequent neurological disability [56]. Both pathogenic Th1 and Th17 responses are believed to be involved in driving inflammation in both MS patients and the animal model experimental autoimmune encephalomyelitis (EAE) while Treg and Th2 cells are thought to play a dominant role in preventing autoimmunity [57,58]. IL-27 has been implicated in the immune pathogenesis of MS and is a potential therapeutic target in CNS autoimmune diseases [8]. Although IL-27 acts as a double-edged sword, as it exerts both immunosuppressive and inflammatory functions in various immune-mediated conditions, the most immunoregulatory effect of IL-27 has been found in different studies in MS/EAE [8]. For example, even though there is much evidence that IL-27 is a potent inhibitor of Th1 cell development in vitro, it is able to constrain the Th1-mediated inflammatory responses in vivo [38,59].

**Figure 2:**
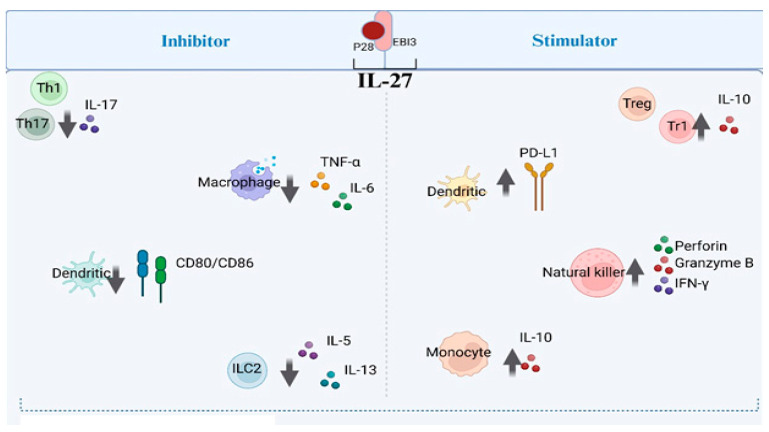
IL-27 reduces inflammation and protects nerve cell in multiple sclerosis (MS) by regulating immune responses. The regulatory effects of IL-27 on various immune cells involved in autoimmune responses. IL-27 influences multiple immune pathways: it inhibits Th1 and Th17 pro-inflammatory responses while promoting Treg and Tr1 anti-inflammatory functions. It modulates dendritic cells by increasing PD-L1 expression and reducing CD80/86, while also reducing pro-inflammatory cytokine production by macrophages and increasing reactive oxygen/nitrogen species. In natural killer (NK) cells, IL-27 enhances proliferation and the production of cytotoxic molecules like granzyme B, perforin, and IFN-γ. It suppresses ILC2 proliferation and decreases IL-5 and IL-13 production. IL-27 also promotes inflammatory cytokine secretion in monocytes while boosting IL-10 production. Collectively, the actions of IL-27 help to control immune-mediated inflammation, offering potential neuroprotective effects in MS. (Created with BioRender.com).

An increasing body of evidence indicates that plasma/serum IL-27 levels were significantly lower in patients with MS as compared with healthy controls, indicating that they may be involved in MS pathophysiology [56,60,61]. In support of this finding, a significant inverse correlation was observed between the level of plasma IL-27 as well as the frequency of Th17 cells in the blood [56]. This inference was supported by the finding that IL-27 suppressed encephalitogenic Th17 responses by downregulation of RORγt and induction of IL-10-producing type 1 regulatory T (Tr1) cells, directly and indirectly, respectively (Figure 2) [62,63]. Furthermore, a study using the EAE mouse model of MS demonstrated that the genetic deletion of the IL-27Ra resulted in exacerbated inflammatory responses and aggravated the severity of EAE, which is associated with increased Th-17 cells production and elevated production of Th17-associated cytokines, including, IL-17, IL-17F, IL-6, and TNF [37]. Furthermore, IL-27 has been shown to attenuate Th17-driven inflammation in models of EAE in vivo [37]. Following systemic IL-27 administration, inflammatory infiltration and demyelination in the CNS were potently reduced, suggesting the neuroprotective activity of IL-27 [62]. Another experiment confirmed the immunoregulatory effect of IL-27 on CNS autoimmunity by showing lower Th17 and Th1 cells within the CNS in IL-27-treated mice compared with PBS-treated control animals [62]. Importantly, in vivo over-expression of IL-27 p28 diminishes autoimmune responses in EAE and experimental autoimmune uveitis by concurrently antagonizing Th1 and Th17 responses [64]. Relevant to the human disease, the protective effect of IFN-β, first-line therapy in relapsing–remitting multiple sclerosis, in patients with MS, was attributable to IL-27 induction, which promotes the production of IL-10 by dendritic cells [65,66]. These important observations suggest that IL-27 may be used as a potential target for the future treatment of MS, but more studies need to be performed to verify these results.

## 6. The Dual Role of IL-27 in Rheumatoid Arthritis: Balancing Inflammation and Immune Regulation

Around 1% of the world’s population is affected by the severe inflammatory autoimmune illness rheumatoid arthritis (RA), which has a high morbidity and mortality rate [67]. It has been demonstrated that IL-27 is expressed in RA synovial tissue and synovial fluid [68,69,70]. In addition, it was shown that RA patients’ serum and synovial tissue had higher levels of IL-27 [70,71].

IL-27 exhibits both anti- and pro-inflammatory properties, according to research on RA and animal models of the condition. In RA joints, it was shown that CD14^+^ mononuclear cells (MNCs) rather than fibroblast-like synoviocytes (FLS) are the main sources of IL-27 production. It appears that these MNCs mediate their anti-inflammatory actions via infiltrating into the inflamed synovium of arthritic joints [70]. According to tests conducted on human osteoclasts, IL-27 can also inhibit osteoclastogenesis. When compared to murine osteoclasts, which express IL-27 receptors at relatively lower levels, human osteoclasts exhibit a significantly stronger inhibitory effect on osteoclastogenesis. This impact was connected to the suppression of the RANK expression, NFATc1 induction, and RANKL-induced nuclear factor-kB (NF-kB) and MAPK pathway activation. Thus, by preventing osteoclastogenesis, IL-27 can prevent bone degradation caused by arthritis [68]. Additionally, supporting its anti-arthritic properties, IL-27 has been demonstrated to decrease T cell RANKL expression [72].

The development of ectopic lymphoid structures, which appear in RA patients’ synovial membranes, is thought to be specifically suppressed by IL-27. Th17 cells and T follicular helper (TFH) cells that express podoplanin are necessary for the development of these lymphoid aggregates. Ectopic lymphoid structures substantially increase in quantity and severity in experimental arthritis in the absence of IL-27 [73]. Collectively, these data indicate that IL-27 balances the composition of the T-cell compartment in the synovial membrane, thereby preventing the formation of lymphatic aggregates.

The disease-protective properties of IL-27 have been elucidated by studies using the collagen-induced arthritis (CIA) model of RA. For instance, in mice with CIA, the local production of IL-27 utilizing intra-articular injections of an adenoviral vector resulted in a decrease in the histological and clinical signs of arthritis. It was discovered that these mice had lower levels of IL-17 in their serum and joints [74]. Similar to this, IL-27 therapies decreased Th17 but raised Foxp3-expressing Treg, changing the Th17/Treg balance towards disease regression in mice with CIA. Additionally, both in vivo and in vitro, IL-27 increased the suppressive function of Treg and Foxp3 expression, and IL-10 production was increased by IL-27. Additionally, PBMC from RA patients showed evidence of the role of IL 27 in altering the Th17/Treg balance [19]. In a different investigation, IL-27 therapies mitigated the severity of arthritis in mice with CIA. This was associated with a decrease in cellular infiltration into the joints, synovial hyperplasia, bone erosion, serum IL-6 levels, collagen-specific IgG2a levels, and peripheral lymphoid cell productions of IFN-γ and IL-17 [69,75].

**Figure 3:**
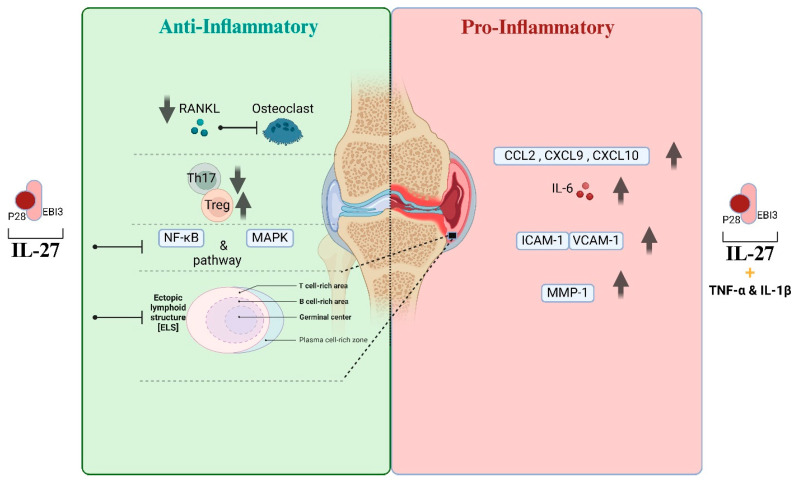
The dual role of IL-27 in rheumatoid arthritis (RA). This diagram illustrates the dual anti-inflammatory and pro-inflammatory effects of IL-27 in RA. On the anti-inflammatory side, IL-27 inhibits RANKL, reducing osteoclastogenesis and, thus, preventing bone degradation. It also shifts the Th17/Treg balance towards Treg cells, suppressing inflammation. Additionally, IL-27 inhibits the NF-κB and MAPK pathways, while preventing the formation of ectopic lymphoid structures (ELS) in the synovium, contributing to reduced immune cell infiltration. Conversely, on the pro-inflammatory side, IL-27, along with TNF-α and IL-1β, induces the expression of IL-6, ICAM-1, VCAM-1, MMP-1, and chemokines, such as CCL2, CXCL9, and CXCL10, which promote inflammatory responses in RA. These factors enhance immune cell recruitment and activation, contributing to joint inflammation and tissue destruction. (Created with BioRender.com).

In contrast to the results discussed above, some of the earlier studies suggested that IL-27 may play a pro-inflammatory function in RA. ICAM-1, VCAM-1, IL-6, chemokines (CCL2, CXCL9, CXCL10), and matrix metalloproteinase-1 (MMP-1) have all been demonstrated to be induced by IL-27 in RA-FLS. Additionally, the pro-inflammatory effects of IL-27 demonstrated synergy with those of TNF-α or IL-1β (Figure 3) [76]. The neutralization of the p28 component of IL-27 by autoantibodies was demonstrated to reduce ongoing adjuvant-induced arthritis (AA) in Lewis rats in a study using the rat AA model [77]. In the Th1-mediated proteoglycan-induced arthritis (PGIA) model of RA, IL-27R (TCCR)-/-mice did not develop the illness. These outcomes were linked to a decrease in IFNγ-producing Th1 cells in these animals, but not in T cells that expressed IL-4 or IL-17. In this model, IL-27 caused Th1 cells to proliferate and increased IFN-γ production, which led to the development of the illness [78].

## 7. The Complex Role of IL-27 in Type 1 Diabetes: From Pathogenesis to Potential Therapeutic Target

Type 1 diabetes (T1D) is a chronic autoimmune disease that leads to the destruction of insulin-producing pancreatic β cells [79]. Research on both humans and the NOD mouse model suggests that CD8 T cells are the main agents of β-cell destruction in T1D. In human T1D patients, CD8 T cells are the most prevalent cell type within the islet infiltrate, and in situ MHC tetramer staining has shown their β-cell reactivity [80,81]. In NOD mice, CD8 T cells are necessary for islet inflammation and T1D development [82,83,84]. After their initial activation in the pancreatic lymph nodes (PLN), autoreactive CD8 T cells travel to the pancreas, recognize their specific antigen in conjunction with MHC class I molecules expressed on the surface of β cells, and initiate cytotoxic programs [85,86,87,88]. However, the specific mechanisms that regulate autoreactive CD8 T cell differentiation and maintain their effector activity during T1D progression are not completely understood. T1D is a polygenic disease, and genome-wide association studies and meta-analyses have identified more than 50 significantly associated loci in humans [89,90,91,92,93,94,95]. Among the list of T1D-related genes, IL-27 (which encodes the p28 subunit) is one of the important genes involved in the T1D pathogenesis [89,96]. Furthermore, human genetic studies have suggested that IL-27 plays an inflammatory role in the development of T1D [97,98]. Research in the non-obese diabetic (NOD) mouse model has shown that IL-27 is produced by activated DCs in diabetic mice, and blocking IL-27 significantly postponed the onset of T1D transferred by splenocytes in lymphocyte-deficient NOD-scid recipients [99].

A two-sample mendelian randomization study indicated that elevated circulating protein levels of EBI3 (a component of IL-27) are linked with an increased risk of T1DM [100]. Moreover, it has been consistently observed that T1DM patients have significantly elevated levels of circulating IL-27 [101]. Mutations in the IL-27 gene in T1DM contribute to immune disorders. Expression quantitative trait loci (eQTL) analyses have shown that mutations in the IL-27 gene (rs181206) lead to an increased expression of interferon regulatory factor 1 (IRF-1) in CD4^+^ T cells, thereby promoting the production of IFN-γ in T1DM patients [98]. On the other hand, a different study where diabetes was induced by multiple injections of low-dose streptozotocin demonstrated that IL-27 signaling provided protection against T1D [102].

**Figure 4:**
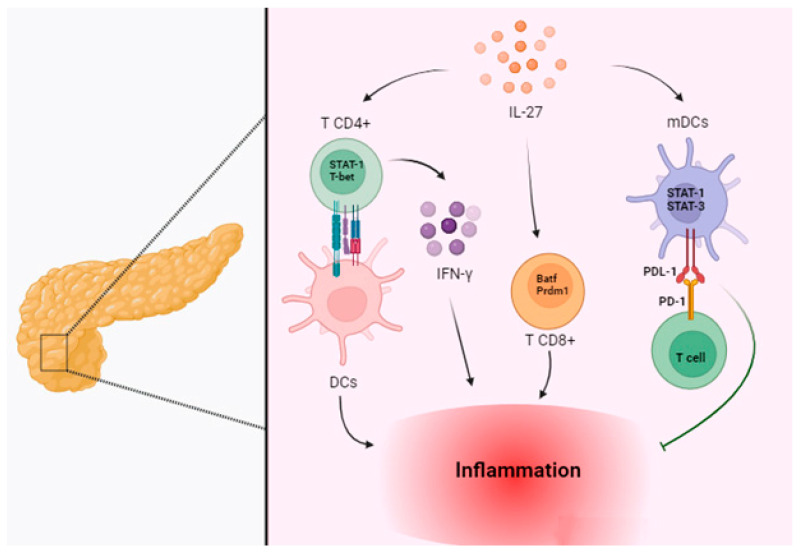
IL-27 and diabetes. In the pancreatic islets, direct IL-27 signaling promoted the growth of T-bet+ CD4 T cells and increased their IFN-γ production. This also suggests that CD4 T cells’ intrinsic IL-27 signaling improves their capacity to activate APCs. IL-27 has the ability to stimulate CD8 T cell responses and upregulate the production of transcription factors, including Batf and Prdm1. All these effects contribute to an increase in inflammation. Conversely, this might imply that IL-27 has a tolerogenic function by raising the phosphorylation of STAT-1 and STAT-3, which leads to an increase in PDL-1 on the surface of myeloid dendritic cells (mDCs) and creates a tolerogenic DC phenotype that helps to reduce inflammation in the pancreatic islets. (Created with BioRender.com).

In a study led by Z. Parackova et al., the researchers found an increased expression and signaling of IL-27 Rα in myeloid dendritic cells (mDCs) of patients with T1D [103]. This was accompanied by increased phosphorylation of STAT1 and STAT3, along with an increased expression of PD-L1 on the surface of mDCs [103]. Additionally, they observed an elevated expression of inhibitory receptors in T1D PBMCs in response to IL-27 stimulation, suggesting a potential role for IL-27 Rα signaling in the tolerogenic compensatory control of T1D (Figure 4) [103].

It has been shown that STAT3 signaling induces a tolerogenic phenotype in DCs, while STAT3 deficiency results in inflammation [104]. This could suggest a tolerogenic role for IL-27 as a STAT3 activator in DCs. Furthermore, IL-27 is known to induce PD-L1 expression in various cell types through STAT1 activation [39,105,106], implying an anti-inflammatory role for IL-27. IL-27 signaling might contribute to their suppressive activities via a STAT1-dependent manner [107,108] or by reducing Th17-mediated inflammation, as demonstrated in an autoimmune model of experimental autoimmune encephalomyelitis [62].

Many of the pro-inflammatory effects of IL-27 signaling are mediated through the activation of STAT1 [109]. The completely protective phenotypes of *NOD.IL27^−/−^* and *NOD.IL27ra*^−/−^ mice are particularly noteworthy given that *NOD.STAT1^−/−^* mice are also completely protected from insulitis and T1D [110]. In contrast, NOD mice deficient in other genes immediately upstream of STAT1 signaling, including IL-6, Ifng, Ifngr2, and Ifnar1, develop insulitis and T1D [111,112,113]. Interestingly, NOD mice deficient in both Ifngr1 and Ifnar1 still develop T1D, although the overall incidence is reduced [114]. Both type I and type II interferon (IFN) pathways can also induce IL-27 expression [115,116,117]. These results collectively suggest that signaling pathways independent of type I and type II IFNs stimulate IL-27 expression, leading to the activation of STAT1-mediated diabetogenic activities. Earlier studies have shown that IL-27 signaling promotes the expression of T-bet and IFN-γ production by CD4 T cells via STAT1-dependent signaling [9,33,118].

A study conducted by Ashley E. Ciecko et al. (2019) aimed to further elucidate the role of IL-27 signaling in the pathogenesis of T1D autoimmunity. The findings indicated that IL-27 signaling fosters the development of insulitis and progression to T1D through various mechanisms [119]. Direct IL-27 signaling facilitated the accumulation of T-bet+ CD4 T cells and amplified their production of IFN-γ in the spleen, PLN, and pancreatic islets [119]. The study demonstrated that without IL-27 signaling, there is minimal DC and T cell infiltration in the islets and scant expression of CD40 on myeloid APC subsets over time. Furthermore, antigen-specific activation of autoreactive CD8 T cells was inhibited in the PLN of IL-27-deficient hosts. These results collectively suggest that in the absence of IL-27 signaling, DC infiltration into the pancreatic islets and antigen trafficking to the PLN are inadequate to activate CD8 T cells and intensify the autoimmune response. This implies that the IL-27 signaling intrinsic to CD4 T cells enhances their ability to activate APCs in the pancreatic islets [119]. In another study, the role of IL-27 in CD8 T cells in T1D was further substantiated when islet inflammation and increased CD8 T cell responses were observed in mice genetically engineered to overexpress IL-27 [120].

In a separate research by Ashley E. Ciecko et al. (2021), they found that IL-27 signaling fostered the expansion of the islet-infiltrating CD44^high^ TCF1^+^CXCR6^−^CD8 T cells and concurrently drove their differentiation into the CD44^high^ TCF1^−^CXCR6^+^ terminal effector population [121]. Thus, IL-27 signaling might stimulate the proliferation of the islet-infiltrating CD44^high^ TCF1^+^CXCR6^−^ CD8 T cells, thereby accelerating their transition to the terminally differentiated state [121]. Interestingly, a recent study analyzing the gene expression profiles of CD8 T cells stimulated with or without IL-27 reported that *Batf* and *Prdm1* were among the transcription factors significantly upregulated in response to IL-27 stimulation, whereas *Tcf7* and *Elk4* were among the transcription factors that were significantly downregulated in response to IL-27 stimulation [122]. Therefore, IL-27 signaling may foster the differentiation of CD8 T cells during the progression of T1D by directly targeting key transcription factors. Previous studies have associated the effect of IL-27 on regulatory T lymphocytes (Tregs) with both pro- and anti-inflammatory roles. Bin Dhuban et al. demonstrated that IL-27 impairs the suppressive capacity of human Treg via the gp130 receptor subunit [123]. Therefore, it is conceivable that the contribution of Tregs to T1D pathology might be influenced by enhanced IL-27 signaling in T1D patients; however, the exact range of effects is still unclear and warrants further investigation.

## 8. The Dual Role of IL-27 in Inflammatory Bowel Disease: Mediator and Inhibitor of Intestinal Inflammation

A group of gastrointestinal illnesses collectively known as “inflammatory bowel disease” (IBD) are identified by intestinal inflammation. The most prevalent of these disorders, which are caused by several factors, are Crohn’s disease and ulcerative colitis. IL-27 is a cytokine that plays a pivotal role in the pathogenesis of inflammatory diseases, such as IBD [124]. The studies showed that the level of IL-27 was increased in people with active inflammatory bowel diseases. Carballeda et al. demonstrated that IL-27 gene expression was enhanced in patients with active ulcerative colitis, and they also discovered that IL-27 mRNA expression was elevated in individuals with active Crohn’s disease [124]. In addition, in the mucosa and muscle tissues of patients with active ulcerative colitis and active Crohn’s disease, the number of cells that produce IL-27 was increased [124]. Furthermore, it was shown that the two IL-27 subunits, IL-27p28 and Epstein–Barr virus-induced protein 3 (Ebi3), were elevated in IBD patients [125,126].

The study that was conducted on the murine transfer colitis model with a deletion in the IL-27ra (a subunit of the IL-27 receptor) demonstrated that non-expression of IL-27ra leads to an increase in Treg differentiation and, as a result, a reduction in colitis and weight loss [127]. Based on these data, it may be concluded that IL-27 can impede the conversion of T cells into Tregs, which will enhance inflammation [127]. Some studies suggested that IL-27 could perform a pro-inflammatory role in the pathogenesis of IBD by inducing the production of cytokines such as IL1-β and IL-6 in the antigen-presenting cells (APCs), which leads to differentiation of TH17 from naïve TCD4^+^ and subsequently the development of colitis [128].

On the other hand, it was discovered that IL-27 can reduce inflammation in IBD. According to research by Sasaoka et al., IL-27 inhibits Th17 differentiation and suppresses the production of inflammatory cytokines like IL-17A, which improves colitis symptoms such as colon length, body weight, the level of necrosis, ulceration, and inflammation [129]. Also, it is indicated that IL-27 induced the development of regulatory T cell type I (Tr1) differentiation which led to production of IL-10 and consequently reduction in the intestinal inflammation [130,131].

**Figure 5:**
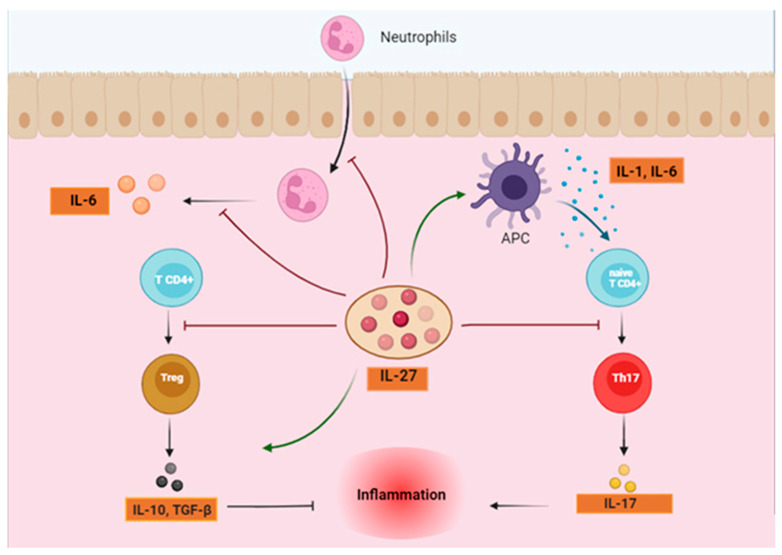
The dual function of IL-27 in the IBD. Antigen-presenting cells (APCs) can be stimulated by IL-27 to release pro-inflammatory cytokines, such as IL-1 and IL-6, which lead to the induction of TH17 differentiation from naïve TCD4^+^ and the subsequent development of disease. In addition, IL-27 can impede the development of regulatory T cells, which contributes to increased inflammation. Conversely, IL-27 can play an anti-inflammatory role in the pathophysiology of IBD by inhibiting the development of TH17 from naïve T cells, which in turn inhibits the production of IL-17; it can also prevent innate immune cells from migrating to the gut and inhibit the production of pro-inflammatory cytokines like IL-6 from neutrophils; Furthermore, it can induce Treg differentiation, which in turn causes the production of anti-inflammatory cytokines, which reduces inflammation. (Created with BioRender.com).

The interaction between IL-27 and IL-27R was shown to influence the CD4^+^ T cell population in the gut, and it was also revealed that Th1 cell frequency was lower and the Th17 pool was higher in mice lacking IL-27R than in WT mice in the colon [132]. Additionally, it was shown that the IL-27-IL-27R interaction prevented the recruitment of innate immune cells to the gut and inhibited the production of pro-inflammatory cytokines like IL-6 from TLR ligand-activated neutrophils, which limited intestinal inflammation [132]. A possible therapeutic method for the treatment of colitis known as LL-IL-27, IL27, which is actively generated in situ by the food-grade bacteria Lactococcuslactis, has been proposed by some studies [133]. This approach increases the production of IL-10 and decreases the expression of pro-inflammatory cytokines like IL-1, IL-6, IFN-γ, IL-23, IL-17A, and IL-17F, which can improve survival and lessen colon and small intestine pathology (Figure 5) [133]. According to this information, IL-27 can act as an inflammatory mediator or an inhibitor in IBD.

## 9. Interleukin 27 in Myasthenia Gravis: Potential Diagnostic Marker and Immunomodulatory Factor

Myasthenia gravis (MG) is a neuromuscular autoimmune disease that can be recognized by various symptoms like muscle weakness and abnormal fatigue. Producing autoantibodies against the acetylcholine receptors, as well as novel targets, including muscle-specific kinase (MuSK) and lipoprotein-related protein 4 (LRP4), have been reported as immunopathology of MG [134].

Different investigations revealed that the serum level of interleukin 27 varies with the disease’s stage, in which its level is significantly higher than that in healthy people at the onset of the disease, which can be used as a diagnostic parameter for MG [135]; However, after treatment with intravenous immune globulin (IVIG), the IL-27 level decreases, probably due to the reduction in T cells that produce interleukin 27 (CD3^+^, IL27^+^ T cells) [136].

The role of IL-27 in the immunopathology of MG has been investigated by a few researchers, in such a way that the effects of IL-27 on naïve T CD4^+^ cause the response to shift towards TH1 and release IFN-γ, IFN-γ affects APCs (the major cells that produce IL-27) and IL-27 is produced again, creating an inflammatory loop of IL-27/IFN-γ; Also, IL-27 by affecting follicular helper T cells (TFH) in the germinal centers, cause the production of interleukin 21, and IL-21 itself causes the production of autoantibodies against acetylcholine receptors and novel targets as it mentions above [134]. IL-27 is known to influence Treg; it is assumed that IL-27 decreases Treg formation.

**Figure 6:**
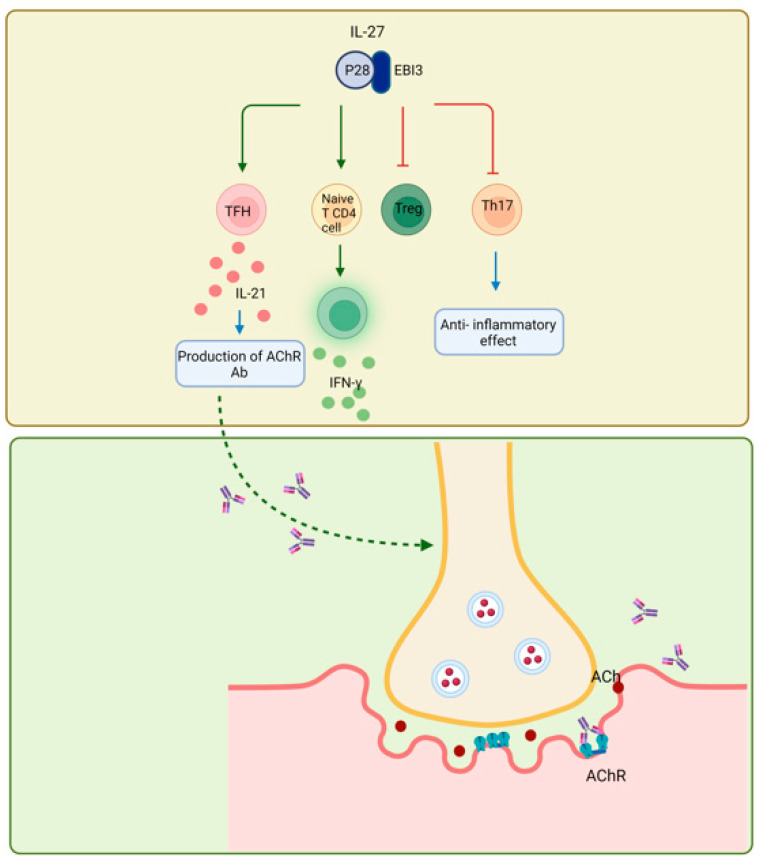
IL-27 plays a crucial role in the immunopathology of MG. The impacts of IL-27 produced by APCs on naïve T CD4^+^ cells shift the response towards TH1 and release IFN-γ. Also, it reduces the production of Tregs. Furthermore, it inhibits the commitment to TH17, which is the anti-inflammatory role of this cytokine in MG. Nonetheless, IL-27 affects TFHs in the germinal centers, causing the synthesis of interleukin 21, and IL-21 itself stimulates the generation of autoantibodies against acetylcholine receptors. Ab, antibody; Ach, acetylcholine; AChR, acetylcholine receptor. (Created with BioRender.com).

The effect of IL-27 on Treg varies depending on the disease; thus, further study is needed to determine the relationship between IL-27 and Tregs in MG patients [135,137]. However, it has been shown that IL-27 suppresses the commitment to Th17, which is this cytokine’s anti-inflammatory role in MG (Figure 6) [134].

Finally, all the statements regarding this cytokine’s impact on the immunopathology of MG are merely hypotheses from various studies on the disease. The increase or decrease in IL-27 serum levels in this situation might only be a manifestation, and no studies specifically targeting IL-27 in these patients have yet been conducted [135].

## 10. Interleukin-27 in Other Autoimmune Disease

Sjögren’s syndrome (SS) is a chronic and multi-systemic autoimmune disease which is identified by the lymphocyte infiltration of exocrine glands [138] (Table 1). Its exact source is unknown, but the immune system’s destruction of the salivary and lacrimal glands is the cause of the disease’s well-known symptoms, xerostomia and xerophthalmia, which correspond to respectably dry mouth and eyes [139]. Systematic SS can also impact other organs, including the vagina, upper respiratory tract, and oropharynx, which can lead to inflammation and highly versatile clinical symptoms in these areas [139]. Studies demonstrated the role of Th17 cells and their cytokine IL-17 in inflammatory and autoimmune diseases [140]. The ratio of Th17 to Treg in maintaining immune homeostasis is very important [141,142]. Thus, IL-27, as a member of the IL-12 family, maintains the balance between Th17 and Treg cells, and the interaction between IL-27 and IL-27R inhibits the differentiation of Th17 cells [8,143]. Studies showed that the level of IL-27 in SS patients decreased; on the other hand, the correlation between serum IL-27 and IgG, unlike IgA and IgM, increased [144]. According to the findings, IL-2,7 as an inhibitor of Th17, may be a useful reagent for treating SS patients; however, generally, the underlying mechanism of IL-27 in SS patients is still unknown [140].

Guillain-Barré syndrome (GBS) is an autoimmune neurological disease, which causes weakness of limbs and areflexia [145]. It is believed that IL-27 is involved in the Guillain-Barré syndrome (GBS), and studies showed that serum IL-27 levels increase during the acute and recovery phase of GBS [146]. IL-27 induces Th1 differentiation, inhibits Th17 cells, and induces IL-10 production, thus suppressing immune responses [147]. Studies showed the induction of the concentration of serum IL-27 in GBS patients and that it probably plays an anti-inflammatory role; however, its main function in pathogenesis of GBS remain unclear [148].

## 11. Conclusions

In conclusion, IL-27 exhibits complex, context-dependent roles in various immune-mediated diseases, acting as both a mediator and inhibitor of inflammation. Understanding its diverse functions opens avenues for targeted therapeutic interventions in autoimmune diseases such as MS, RA, T1D, IBD, Sjögren, and GBS. Further research is needed to elucidate the precise mechanisms and therapeutic potential of IL-27 in these conditions.

## Figures and Tables

**Table 1:** A summary of the evidence-based involvement of IL-27 in autoimmune diseases.

Autoimmune Disease	Circumstance	Protective Effects	Deleterious Effects
Multiple Sclerosis (MS)	Immune-mediated CNS inflammation	Inhibits Th1 and Th17 responses; promotes Treg and Tr1 cells; reduces inflammatory infiltration and demyelination; attenuates CNS autoimmunity in animal models	May contribute to inflammation in specific contexts
Rheumatoid Arthritis (RA)	Joint inflammation and autoimmunity	Decreases osteoclastogenesis; shifts Th17/Treg balance towards Tregs; prevents ectopic lymphoid structures; reduces inflammatory cytokines in animal models	Induces pro-inflammatory molecules (e.g., IL-6, MMP-1) in fibroblast-like synoviocytes; synergizes with TNF-α
Type 1 Diabetes (T1D)	Autoimmune destruction of pancreatic β cells	Activates tolerogenic signaling pathways in dendritic cells; induces regulatory markers like PD-L1	Promotes Th1 and CD8 T cell responses; facilitates IFN-γ production; associated with disease progression
Inflammatory Bowel Disease	Chronic intestinal inflammation	Reduces Th17 differentiation and IL-17 production; promotes Treg and Tr1 cells; prevents innate immune cell recruitment to the gut	Induces pro-inflammatory cytokines (e.g., IL-6, IL-1β); limits Treg differentiation
Myasthenia Gravis (MG)	Autoantibody-mediated neuromuscular dysfunction	Suppresses Th17 differentiation	Promotes Th1 and follicular helper T cell responses; drives autoantibody production; reduces Treg formation
Sjögren’s Syndrome (SS), also known as Sjögren’s Disease (SD)	Lymphocyte infiltration of exocrine glands	Inhibits Th17 differentiation; may help balance Th17/Treg ratio	Mechanisms not well understood; linked to reduced IL-27 levels
Guillain-Barré Syndrome	Autoimmune neurological disease	Induces IL-10 production; inhibits Th17 cells and promotes anti-inflammatory Th1 responses	Main role in pathogenesis unclear; increases IL-27 levels during acute and recovery phases

## Data Availability

Not applicable.
